# Brenner Tumor of the Ovary: A 10-Year Single Institution Experience and Comprehensive Review of the Literature

**DOI:** 10.3390/medsci11010018

**Published:** 2023-02-07

**Authors:** Ferial Alloush, Hisham F. Bahmad, Brendan Lutz, Robert Poppiti, Monica Recine, Sarah Alghamdi, Larry E. Goldenberg

**Affiliations:** 1 Arkadi M. Rywlin M.D. Department of Pathology and Laboratory Medicine, Mount Sinai Medical Center, Miami Beach, FL 33140, USA; 2 Department of Obstetrics and Gynecology, Mount Sinai Medical Center, Miami Beach, FL 33140, USA; 3 Department of Translational Medicine, Herbert Wertheim College of Medicine, Florida International University, Miami, FL 33199, USA

**Keywords:** Brenner tumor, ovary, Walthard rests, mucinous cystadenoma, case series

## Abstract

Brenner tumors (BTs) are surface-epithelial stromal cell tumors that are categorized by the World Health Organization as benign, borderline, and malignant. Due to the rarity of BTs, the published literature on these tumors is comprised primarily of case reports and small retrospective studies. We performed a pathology database review spanning the last ten years at our institution revealing nine reported benign BTs. We collected the clinical and pathological data of patients associated with those BTs, describing the clinical presentation and imaging results, and assessing the possible risk factors associated with them. The average age at diagnosis was 58 years. BTs were discovered incidentally in 7/9 cases. The tumor was multifocal and bilateral in 1/9 cases and ranged in size from 0.2 cm to 7.5 cm. Associated Walthard rests were found in 6/9 cases and transitional metaplasia of surface ovarian and/or tubal epithelium was found in 4/9 cases. One patient had an associated mucinous cystadenoma in the ipsilateral ovary. Another patient had an associated mucinous cystadenoma in the contralateral ovary. In conclusion, we found that Walthard rests and transitional metaplasia are common findings in association with BTs. Additionally, pathologists and surgeons need to be aware of the association between mucinous cystadenomas and BTs.

## 1. Introduction

Brenner tumors (BTs) are surface-epithelial stromal cell tumors that account for 1–5% of epithelial ovarian tumors [1] and 1.4–2.5% of all ovarian tumors [2,3]. They are categorized by the World Health Organization as benign, borderline (proliferative), and malignant [4]. Most tumors are benign or borderline with malignant tumors accounting for less than 5% of cases [5]. They occur most commonly in postmenopausal women with borderline and malignant tumors typically occurring later in life (mean age of 50 years for benign tumors and 60 years for borderline and malignant tumors) [6].

These tumors are usually identified incidentally during imaging studies or surgery in post-menopausal women. Histologically, BTs are characterized by well-circumscribed nests of transitional epithelium surrounded by a fibromatous background [2].

It is hypothesized that BTs arise from cells of the tuboperitoneal junction which undergo transitional cell metaplasia before invaginating into the paratubal or ovarian surface, forming Walthard rests. It is postulated that these rests may eventually seed the ovary and develop into BTs, although the mechanism has not been elucidated [7]. Owing to shared morphologic features of benign and proliferative BTs, it is generally believed that the latter can develop from the former [8] through a metaplastic pathway from the ovarian surface epithelium or stromal tissue. Such a pathway makes possible the development of morphologically similar proliferative and malignant BTs, which are far rarer compared to benign BTs. Less than 5% of BTs are borderline [9] and, as of 2019, fewer than 60 cases of borderline BTs had been reported [1].

Research by Kuhn et al. [8] suggested that a genetic loss of *CDKN2A*, a p16-encoding gene, and to a lesser extent *KRAS* and *PIK3CA* mutations, may play a role in benign BTs progressing to atypical, proliferative Brenner tumors. Perhaps due to the rarity of BTs and the non-malignant character of their majority, the published literature on these tumors is primarily comprising case reports and small retrospective studies. The study of additional cases of benign BTs may serve to better characterize the patients who are likely to develop these tumors. A comprehensive pathology database review spanning the last ten years at our institution revealed benign BTs identified in nine patients and no borderline or malignant BTs reported. Objectives of our study include: (1) collecting the clinical and pathological data of patients with a diagnosis of BT of the ovary treated at our institution during the past decade, (2) describing the clinical presentation and imaging results, and (3) assessing possible risk factors associated with BT.

## 2. Materials and Methods

### 2.1. Study Design, Setting, and Objectives

The pathological database of surgical specimens from patients with Brenner tumor of the ovary during a ten-year period, from 1 July 2012 to 31 August 2022, at Mount Sinai Medical Center (Miami Beach, FL, USA) was reviewed. This study represents a case series analysis with no direct physical risk to the patients in the study. Chart review was performed, and patient information was collected.

### 2.2. Ethical Considerations

Approval of the Institutional Review Board (IRB) of Mount Sinai Medical Center of Florida was granted prior to commencement of the study. All protocols followed in our retrospective cohort study were performed in accordance with guidelines and regulations of The Code of Ethics of the World Medical Association (Declaration of Helsinki). The study was performed in a manner that ensures confidentiality of patients.

### 2.3. Patients’ Selection

Inclusion criteria included patients who underwent oophorectomy or other ovarian sampling procedures during the time-period specified. No exclusion criteria were present.

### 2.4. Clinicopathological Parameters of Patients

Clinical and pathological parameters of patients were retrospectively retrieved from electronic medical records, including age, clinical data (signs and symptoms, imaging results), focality (bilateral or unilateral), side (right or left), site, type of surgery, indication of surgery, risk factors, pathological diagnosis, and features (presence of Walthard rests and transitional metaplasia), and follow-up data.

## 3. Results

A 10-year retrospective search of the pathology database at our institution showed nine cases of Brenner tumor (BT). Age at the time of diagnosis ranged from 34 years to 76 years with an average of 58 years. All cases were benign in nature. Seven of the nine cases were incidentally discovered during radiological and surgical assessment for other conditions (Supplementary Table S1). The remaining two symptomatic cases presented abdominal distension and abdominal pain. Six of the nine cases had a normal intra-operative appearance of the involved ovary. Pre-operative CA-125 was within the reference range in four of the nine cases and was unknown in the remaining five patients (Table 1).

The tumor was multifocal and bilateral in one of the nine cases and ranged in size from 0.2 cm to 7.5 cm with five of the nine cases measuring less than 1 cm in maximum dimension, which explains the incidental mode of presentation in most of our cases. Associated Walthard rests were found in six of the nine cases. Transitional metaplasia of surface ovarian and/or tubal epithelium was found in four of the nine cases. Additionally, one patient had an associated 30 cm mucinous cystadenoma in the ipsilateral ovary. Another patient had an associated 17 cm mucinous cystadenoma in the contralateral ovary (Table 2); the ovary with BT appeared normal intra-operatively. The mucinous component was predominant in both cases.

Of note, a review of our pathology database for the same period (10 years) revealed 23 cases of mucinous cystadenoma; thus, 4% of all resected mucinous cystadenoma at our institution had an associated ipsilateral benign BT, increasing to 9% when accounting for contralateral tumors. One patient had endometrial hyperplasia. Another patient had an associated 8.2 cm corpus luteum cyst in the same ovary where a minute BT was discovered incidentally in association with the cyst. Two of the patients (case #2 and case #5) had a remote history of breast cancer; however, no further data on their breast cancer diagnosis and pathology were available. One of these two patients was *BRCA1* positive.

## 4. Discussion

Brenner tumors (BTs) of the ovary make up approximately 5% of all epithelial ovarian neoplasms [10].

Most BTs of the ovary are unilateral. The percentage of bilateral tumors ranges between 3.7% and 8% in various case series [11,12,13]; 11% in our study. Malignant tumors are more likely to occur bilaterally, 12.6% in a 207 patients population-based analysis [14]. Benign BTs are asymptomatic and are typically found incidentally during imaging studies and surgeries performed for other purposes [2]. Borderline and malignant BTs on the other hand most commonly present with symptoms including abdominal pain, pelvic pressure or less frequently, abnormal uterine bleeding [1,5,15].

**Figure 1:**
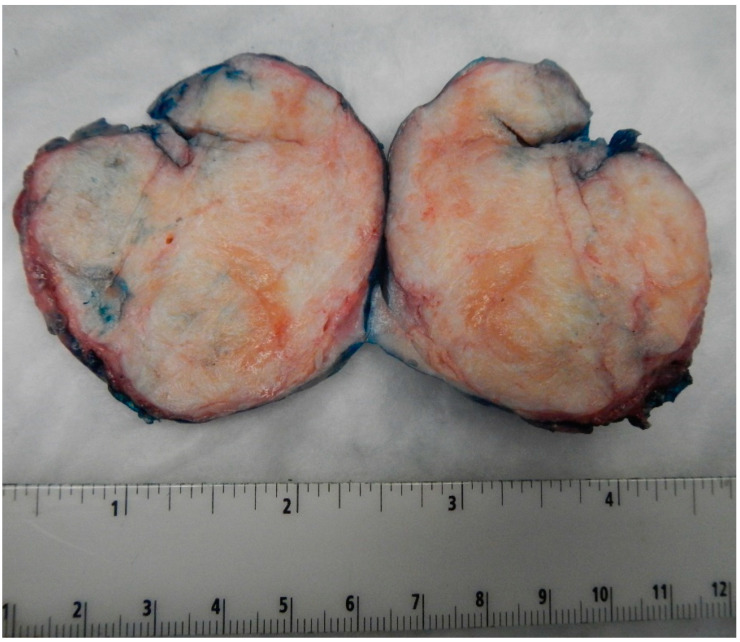
Image of the largest tumor in our cases series measuring 7.5 cm in maximum dimension. Notice the well-circumscribed outlines of the tumor and the tan-white firm cut surface, like those seen in ovarian fibromas.

Benign BTs of the ovary are typically solid and well-circumscribed grey-white nodules (as shown in Figure 1 from our patient population), measuring less than 2 cm in most cases [16]. However, large tumors have been reported with the largest tumor reported in the literature measuring 39 cm in the maximum dimension [16]. In our case series, the largest tumor size was 7.5 cm. Borderline and malignant tumors are larger with a median size of 12 cm and 10 cm, respectively [14,17]. They are typically multilocular or unilocular solid and cystic tumors with variable solid nodules and papillary excrescences in the cystic parts [1,14,18]. Thus, gross and radiologic differentiation from other epithelial ovarian neoplasms may be challenging.

**Figure 2:**
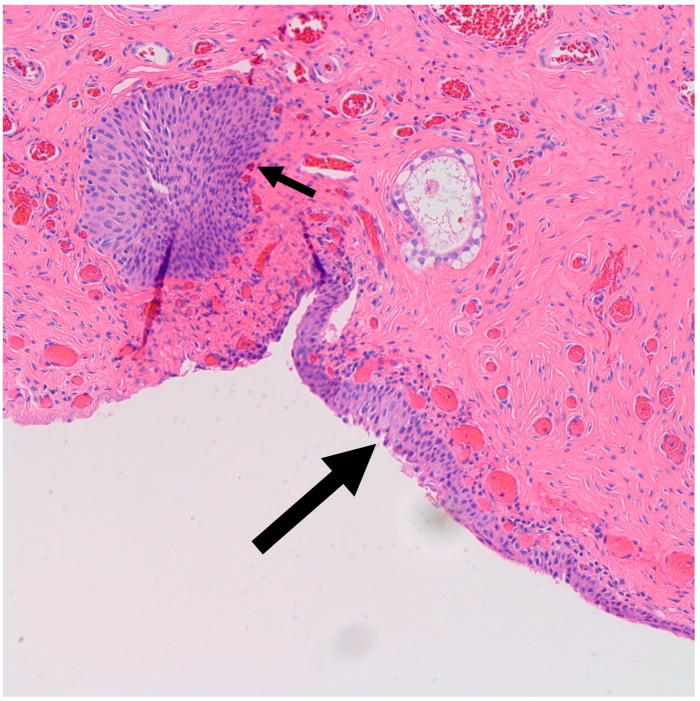
H&E microscopic image of transitional metaplasia (Large arrow) of surface ovarian epithelium with adjacent Walthard rest (Small arrow) (200× magnification).

It is postulated that BTs arise from Walthard rests—rests of metaplastic transitional epithelium—commonly encountered in the fallopian tubes and ovarian hilum [19,20]. Although one study demonstrated the presence of Walthard rests in 30% of BTs compared to 28% in control subjects, authors concluded that the association is weak given the possibility that it was confounded by more extensive sampling [19]. Interestingly, similar to BT, Walthard rests are positive for GATA3 by IHC while negative for PAX-2, PAX-8 and WT-1 [21]. In our study, Walthard rests were present in six out of nine cases (67%) (Figure 2). This suggests that Walthard rests formation is the main predisposing factor for developing BTs. Further research is necessary to better understand the histogenesis of BTs.

**Figure 3:**
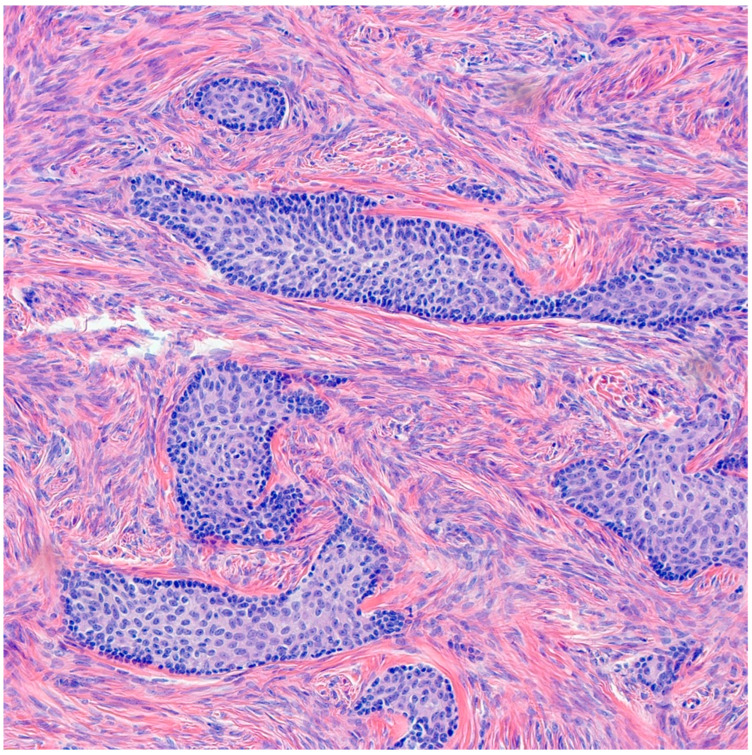
H&E microscopic image of benign Brenner tumor. Notice the nested architecture and the monotony of transitional epithelium. Additionally, note the dense fibromatous background (200× magnification).

**Figure 4:**
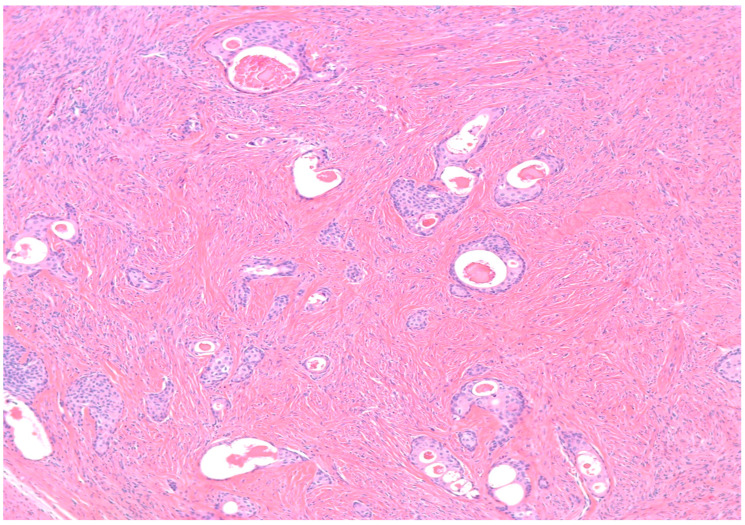
H&E microscopic image of extensive cystic changes involving the epithelial nests of a benign Brenner tumor (100× magnification).

Benign BTs are composed of transitional epithelium with occasional longitudinal grooves arranged in a nested pattern in a prominent fibromatous background (Figure 3). Cystic changes with mucinous and ciliated metaplasia of the luminal epithelial layer are not uncommon [18] (Figure 4). These changes are reminiscent of cystitis cystica and cystitis cystica et. glandularis commonly found in Von-Brunn’s nests of urothelium-lined mucosal surfaces. Borderline BTs show papillary urothelial proliferation reminiscent of that seen in low grade papillary urothelial carcinoma [18]. Metaplastic changes, complex gland formation, moderate to severe cytological atypia, necrosis and increased mitotic activity can be seen [22,23,24,25]. There should be no stromal invasion [18]. A concurrent benign component is present in most borderline cases [18]. Malignant BTs have stromal invasion characterized by a desmoplastic response in the fibromatous background, each case should have a concurrent benign component present [15]. Absence of a benign component should point to the diagnosis of high grade serous carcinoma with solid, pseudo-endometrioid and transitional cell carcinoma-like features [26].

**Figure 5:**
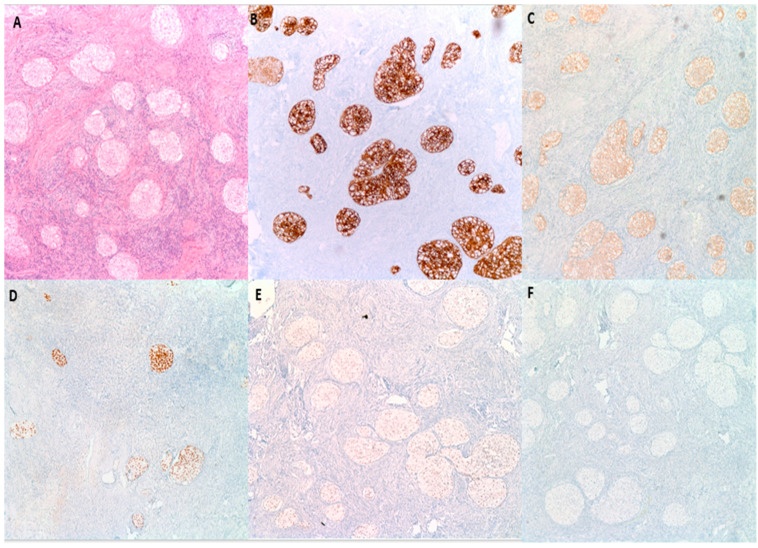
Immunohistochemical characteristics of benign BTs. (**A**) H&E microscopic image of a benign BT. (**B**) Tumor cells are diffusely and strongly positive for CK7. (**C**) CK20 shows moderate intensity staining. (**D**) P63 is diffusely and strongly positive. (**E**) GATA3 shows moderate intensity nuclear reactivity. (**F**) PAX8 is negative (100× magnification).

By immunohistochemistry (Figure 5), BTs are positive for S100 p in 88% of cases, GATA3 in 96% of cases and P63 in 100% of cases [27]. They are also typically positive for CK7, CK20, Uroplakin III, AR (Androgen receptor) and AKR1C while negative for PAX2 and PAX8 (markers of mullerian differentiation), Calretinin and inhibin (markers of stromal ovarian neoplasms), and SALL4 (a germ cell tumor marker) [7,28]. This IHC profile highlights the similarity to urothelial epithelium [27]. BTs are reactive for EGFR, RAS, and Cyclin D1 in a pattern that parallels their degree of aggression. Benign tumors are typically weakly positive for these three markers; borderline tumors are moderately positive and malignant tumors are strongly positive [29]. P53 on the other hand is negative [29]. P16 is typically positive in benign BTs, while negative in borderline and malignant BTs, while [8,26]. EGFR, RAS, Cyclin D1, P16 and P53 are particularly helpful when trying to distinguish the histologically similar malignant BT from high grade serous carcinoma with solid, pseudo-endometrioid and transitional cell carcinoma-like features, as the latter is usually positive for P16 and P53 while negative for EGFR, RAS, and Cyclin D1 (Table 3) [29].

Loss of function of *CDKN2A*, a tumor suppressor gene that encodes P16, by promoter hypermethylation or homozygous deletion has been implicated in the progression of benign to borderline BTs [8]. This is further supported by the fact that most borderline BTs have an associated benign counterpart. P16 can be used as a surrogate marker for loss of *CDKN2A* as the intensity of P16 immunohistochemical staining is directly proportional to the number of CDKN2A copies [8]. Deregulation of EGFR-Ras-Raf signaling pathway, hypermethylation of Rb tumor suppressor gene and *PIK3CA* mutations have also been reported [29].

The differential diagnosis for BTs includes the following:

- Walthard cell rests: The differential diagnosis of benign BTs includes Walthard cell rests. Benign BTs contain a fibromatous background which is absent in Walthard cell rests.
- High-grade serous carcinoma (HGSC) with solid, pseudo-endometrioid, and transitional cell carcinoma-like features (SET features): The differential diagnosis of malignant BTs includes HGSC with SET features (formerly known as transitional cell carcinoma of the ovary). Previously, the World Health Organization (WHO) divided malignant transitional cell tumors into transitional cell carcinoma (TCC) non-Brenner type and malignant BT with the distinction based on the presence of a benign Brenner component in the latter and its absence in the former [30]. However, in 2012 Ali et al. showed in their research on a large cohort of ovarian neoplasms which included seven “TCCs” that similar to HGSC, transitional cell carcinoma of the ovary is predominantly positive for ER, PR and P53 by immunohistochemistry [26]. P16^INK4a^ expression was only detected in 2 out of 7 cases [26]. However, the authors suggested that their use of tissue microarrays and the small tumor sample size had resulted in underestimation of the actual frequency of P16 expression in “TCC” which is usually patchy in nature [26]. Additionally, Riedel et al. showed in their research that BTs tend to be positive for urothelial markers including CK20 and Uroplakin III [28]. “TCCs” on the other hand are negative for these two markers and are therefore lacking true urothelial differentiation [28]. Thus, in the latest WHO classification TCC of the ovary was eventually lumped under the category of “HGSC with SET features”, a morphological variant of HGSC commonly seen in BRCA1/2 mutant population [31]. HGSC with SET features and BT exhibit significantly different biological behaviors with HGSC with SET features more commonly having extra-ovarian spread at initial presentation. Hence, patients are more likely to benefit from adjuvant chemotherapy and the distinction is crucial [15]. The presence of a benign Brenner component is diagnostic of malignant BT while absent in HGSC with SET features [15,29]. By immunohistochemistry, HGSC with SET features is usually positive for P53 and P16 while negative for EGFR, RAS, and Cyclin D1 [29]. Malignant BT, being negative for P53 and P16 while positive for EGFR, RAS, and Cyclin D1 exhibits the exact opposite immunohistochemical profile [29]. Genetically, malignant BT arises from precursor benign and borderline components by upregulation of the EGFR signaling pathway which activates downstream cytoplasmic proto-oncogene proteins including RAS -MAPK and PIK3CA-AKT [29]. HGSC with SET features on the other hand arises de novo by *P53* mutation [29].
- Metastatic urothelial carcinoma: Although metastatic urothelial carcinoma to the female gynecological tract is exceedingly rare, it has morphological and immunohistochemical overlaps with malignant BT [28,32]. However, the presence of a benign component is characteristic of BT [15,29]. Extensive sampling may be necessary to identify the benign component. Additionally, clinical and radiological correlation is particularly helpful in this context [32].
- Adult granulosa cell tumor (AGCT): Due to the presence of longitudinal grooves, AGCT—a sex cord-stromal tumor—can be confused with BT. However, diffuse and trabecular patterns are the most common architectural patterns seen in AGCTs while a nested pattern is the norm in BTs [33]. Additionally, AGCTs commonly undergo luteinization [34]. By immunohistochemistry, AGCTs are typically positive for calretinin, inhibin and SF1, all of which are expected to be negative in BTs [35]. By molecular genetics, AGCTs are characterized by somatic missense mutations in the transcription factor FOXL2 gene [36].

It has been reported that BT of the ovary is associated with mucinous neoplasms, most commonly mucinous cystadenoma. Mucinous cystadenoma is one the most common benign epithelial neoplasms of the ovary and is the precursor of mucinous carcinoma. Mucinous ovarian neoplasms exhibit a spectrum of differentiation (Table 4) with most tumors (71%) showing gastrointestinal immunophenotype (CLDN18+/CDX2±/ER−), 6% showing pure mullerian differentiation (CLDN18−/CDX2−/ER+) and the remaining cases exhibiting dual gastrointestinal and mullerian differentiation (CLDN18+/CDX2±/ER+) [37].

**Figure 6:**
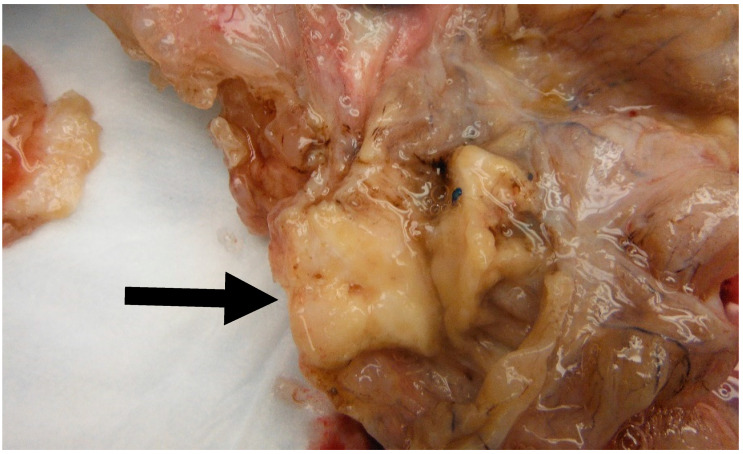
Image of a mural solid nodule (Black arrow) measuring 3.5 cm found in a 30 cm mucinous cystadenoma. Note the solid appearance of the nodule as opposed to the cystic configuration of the mucinous component.

**Figure 7:**
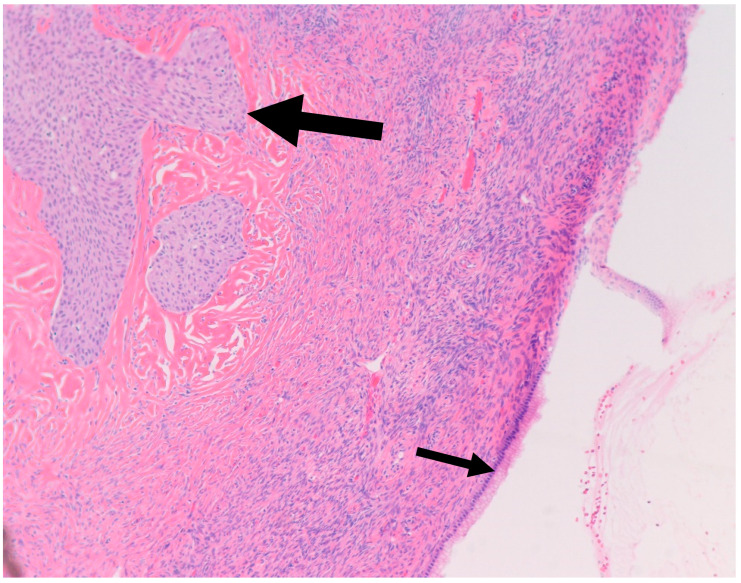
H&E microscopic image of a Brenner tumor (Large arrow) found in the wall of a mucinous cystadenoma (Small arrow). Notice the close proximity between the two components (100× magnification).

**Figure 8:**
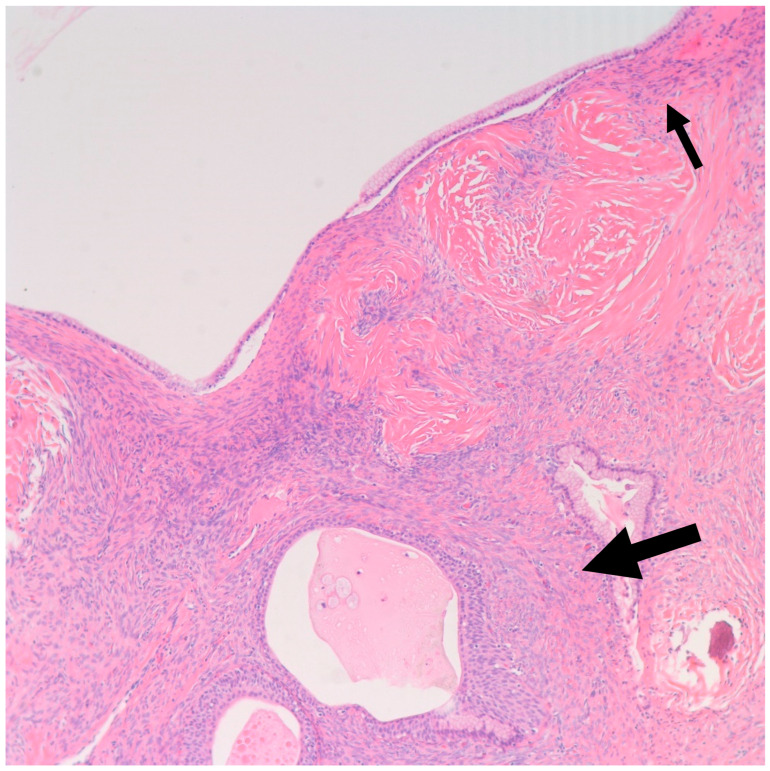
Shown is a 100× magnification H&E microscopic image of mucinous metaplasia of a nest of Brenner tumor (Large arrow) with an adjacent mucinous cystadenoma (Small arrow) (100× magnification).

In a literature review that included 460 Brenner tumors, Waxman et al. reported an associated ipsilateral mucinous tumor in 14.3% of cases, increasing to 17%, when contralateral mucinous tumors were reported [38]. Other smaller studies reported a higher percentage of association reaching up to 30% [21,39]. This higher percentage may be attributed to small areas of mucinous metaplasia commonly found in BTs that might be clinically insignificant [40]. In our study, we report an associated ipsilateral mucinous cystadenoma in 11% of cases, increasing to 22%, when accounting for contralateral tumors. Conversely, 4% of all resected mucinous cystadenoma at our institution showed an associated ipsilateral Benign BT, increasing to 9% when accounting for contralateral tumors. Benign BTs are composed of nests of transitional epithelium with longitudinal grooves in a fibromatous background. Central cystic changes with mucinous metaplasia of the luminal aspect are common findings. Hence, it was postulated that overgrowth of the mucinous component accounts for the common association between BTs and mucinous cystadenomas [41]. On the other hand, it is uncommon for mucinous lesions to show transitional metaplasia [19]. However, this might be confounded by the relatively small size of BTs when compared to the mucinous component [19]. In fact, they can be easily missed due to inadequate sampling. Mucinous tumors and BTs tend to be negative for PAX-2 and PAX-8, two highly sensitive mullerian epithelial markers that are often positive in other ovarian epithelial neoplasms [40,41]. This further suggests shared histogenesis of these two lesions [40,41]. Addressing the genetic aspect, in their study of six BTs associated with a mucinous tumor, Tafe et al. showed that four of the six tumor pairs showed *KRAS* hotspot driver mutations in the mucinous component [7]. *MYC* amplification was detected in both mucinous and Brenner components of the two paired samples that lacked *KRAS* mutations [7]. In their study of 10 BTs associated with a mucinous neoplasm, Wang et al. showed a concordant X-chromosome inactivation pattern between the two components in all cases, proving a shared clonal origin [41]. An associated mucinous cystadenoma was present in the ipsilateral ovary in one of our cases. Of note, the mucinous component was predominantly presenting as a 30 cm cyst with a 3.5 cm mural nodule representing a Brenner component (Figure 6 and Figure 7). This highlights the importance of thorough examination and sampling of mucinous cystadenomas. Interestingly areas of mucinous metaplasia were found in the Brenner component (Figure 8). This further supports the theory that mucinous cystadenomas represent an overgrowth of a focus of mucinous metaplasia in a benign BT rather than a separate synchronous tumor. Another case in which BT was found incidentally measuring 1.3 cm was associated with a symptomatic contralateral 17 cm mucinous cystadenoma. Notably, six of our nine cases of BT had a normal intra-operative appearance of the involved ovary; this suggests that the normal intra-operative appearance of the contralateral ovary in mucinous cystadenoma cases does not exclude the possibility of BT and bilateral oophorectomy may be the warranted, when feasible.

Of note, two of our patients had a remote history of breast cancer; however, no further data on their breast cancer diagnosis and pathology were available. One of these two patients was *BRCA1* positive. Both tumors were incidental, benign, and sub-centimetric in size. Literature review revealed one case of malignant BT in association with germline *BRCA1* mutation and another case in association with germline *BRCA2* mutation [42,43]. Further research is needed to investigate the role of germline *BRCA 1/2* mutations in the progression of benign BTs.

Excessive androgen production resulting in virilization has been reported in borderline BTs [7], which can have variable clinical presentations such as hirsutism or abnormal uterine bleeding (AUB) [44]. AUB can result from endometrial hyperplasia due to stimulation by estrogens from aromatase-mediated conversion of androgen excess [44]. Patients developing hirsutism are at risk of other estrogen/testosterone mediated effects including breast cancer [44]. One of our cases had concurrent endometrial hyperplasia without atypia. Of note, the Aldo-keto reductase AKR1C3—an androgen producing enzyme—and androgen receptor (AR) are commonly expressed in BTs [20,45].

Ultrasonography, computed tomography (CT), and magnetic resonance (MR) imaging of BTs may demonstrate solid ovarian masses with or without cystic components [46,47,48]. Benign BTs typically grow to be less than 2 cm in diameter while the borderline and malignant subtypes may grow larger than this [16]. One case series of BTs reported that 8 of 17 tumors were not visualized on sonogram and 5 of 14 were not visualized on CT scan [49]. The majority of BTs occur unilaterally and scattered punctate calcifications are a common finding on CT and sonographic scans [47,48]. While mild to moderate enhancing calcifications in the solid components of BTs are typical on CT scan [48], this imaging is not diagnostic. Following enhancement on CT imaging, benign BTs are reported to be slightly enhanced and the borderline component of BTs are moderately or highly enhanced [48]. While multiple characteristic imaging patterns have been identified, BTs have a non-specific gross appearance and may not produce specific characteristic findings on imaging [2], making their diagnosis with such modalities difficult. However, a small retrospective study [46] of BTs reported that extensive calcification in a solid mass or solid component in a multilocular cystic mass was a characteristic finding on CT and MRI. Increased (moderate to high) fluorodeoxyglucose uptake can be seen on positron emission tomography (PET) imaging with borderline and malignant BTs compared to the mild uptake seen with benign BTs [50].

A literature review demonstrated numerous cases [51,52,53,54] in which Meigs syndrome, characterized by a triad of a benign ovarian (fibroma, Brenner, or granulosa cell) tumor, pleural effusion, and ascites [52], is associated with an elevated serum CA-125 level. However, few cases [28,53,54] involved confirmed BTs. One study [28] involving the immunoprofiling of BTs reported the focal presence of CA-125 in some BTs. Additionally, a case report [53] described a patient with bilateral BTs associated with a serum CA-125 of 759 U/mL. Given the paucity of reported cases, CA-125 is not a reliable diagnostic IHC staining marker for BTs. There does not appear to be a specific marker for BTs [3].

In managing BTs, isolated benign BTs are often small and of minimal clinical significance following resection. Symptoms are usually non-specific. However, in symptomatic cases, may include abdominal distension, abdominal or pelvic pain secondary to mass effect, a palpable adnexal mass, or uterine bleeding (in the rare event of an estrogen-secreting BT) [55,56,57]. BTs are associated with endometrial hyperplasia in 4–14% of cases [57] due to testosterone and estrogen release. However, most BTs are asymptomatic and may be discovered incidentally [48]. These tumors may be found inside larger ovarian masses and identified incidentally as a component of the larger mass during histologic study. Approximately, 30% of BTs are identified as one component of a combined lesion [55].

Benign, borderline, and malignant BTs are all treated, at least in part, with surgical resection. Resection typically is curative of any symptoms they may cause and helps obtain pathologic diagnosis. Borderline and malignant BTs can then be staged with the FIGO staging system. Most borderline BTs are stage I [1,17]. While malignant BTs can potentially metastasize, this is rare and a standard treatment for metastatic BTs has not been developed [2]. Uzan et al. described a single reported case of a fatal recurrent borderline BT attributed to incomplete initial resection performed 50 months prior to the patient’s death [17]. Malignant BTs are rare, thus adjuvant chemotherapy and radiation therapy have not been well-studied or standardized, though one study reported a positive response to adjuvant platinum-taxane treatment following complete surgical resection of the malignant BT in a cohort of ten patients [5].

## 5. Conclusions

Most ovarian BTs are benign incidentally detected tumors. Walthard rests and transitional metaplasia are commonly associated findings. Pathologists and surgeons need to be aware of the common association between mucinous cystadenomas and BT as small nodules of BT can be easily overlooked due to the large size and cystic consistency of mucinous cystadenomas in most cases. More research is needed to further understand the histogenesis of BTs, to characterize the patients who are likely to develop these tumors and to investigate the role of germline *BRCA 1/2* mutations in their progression.

## Figures and Tables

**Table 1:** Clinical characteristics of all Brenner tumor patients included in our study.

Case	Age	Presentation	Presenting Symptom	Indication for Surgery	Type of Surgery	Intraoperative Appearance of BT Ovary	Pre-op CA-125 (U/ML)
1	55	Incidental	Post-menopausal bleeding	Bilateral ovarian cysts on imaging	Laparoscopic BSO + Hysteroscopic polypectomy	Normal	9.2
2	60	Incidental	Asymptomatic	Prophylactic surgery (BRCA1)	TAH + BSO	Normal	NA
3	54	Non-incidental	Abdominal distension	Right adnexal mass	Exploratory laparotomy + TAH + BSO	Large right abdominopelvic mass	NA
4	60	Non-incidental	Abdominal pain	Left adnexal mass	TAH + BSO	Enlarged solid left ovary	NA
5	70	Incidental	Urinary incontinence	Cervical prolapse	TVH + BSO	Normal	NA
6	51	Incidental	AUB	Endometrial hyperplasia and fibroids	TAH + BSO	Normal	NA
7	76	Incidental	Recurrent UTI	Right adnexal mass on imaging	Robotic BSO	Normal	7.9
8	34	Incidental	Abdominal pain	Right adnexal mass on imaging	Robotic right oophorectomy	Right ovarian cyst	5.1
9	59	Incidental	Abdominal pain and increased abdominal girth	Left adnexal mass	TAH + BSO + Appendectomy	Normal	7.9

Abbreviations: AUB: Abnormal uterine bleeding; BSO: Bilateral salpingo-oophorectomy; L: Left; R: Right; TAH: Total abdominal hysterectomy; TVH: Total vaginal hysterectomy.

**Table 2:** Pathological characteristics of all Brenner tumor patients included in our study.

Case	Laterality	Size (cm)	Type	Focality	Other Findings	Walthard Rests
1	L	2	Benign	Unifocal	Endometrial polyp	Present
2	R	0.5	Benign	Unifocal	Leiomyomata uteri	Absent
3	R	3.5	Benign	Unifocal	Mucinous cystadenoma, ipsilateral (30 cm)	Present
4	L	7.5	Benign	Unifocal	Leiomyomata uteri	Absent
5	R	0.4	Benign	Unifocal	None	Present
6	R and L	0.5 and 0.2	Benign	Two foci	Leiomyomata uteri	Present
7	R	0.7	Benign	Unifocal	None	Present
8	R	0.2	Benign	Unifocal	Corpus luteum cyst, ipsilateral (8.2 cm)	Absent
9	R	1.3	Benign	Unifocal	Mucinous cystadenoma, contralateral (17 cm) + Endometrial polyps	Present

**Table 3:** Immunohistochemical characteristics of BTs and high-grade serous carcinoma.

IHC Marker	EGFR	RAS	Cyclin D1	P16	P53
Benign BT	Weakly positive	Moderately positive	Strongly positive	Positive	Wild type
Borderline BT	Weakly positive	Moderately positive	Strongly positive	Negative	Wild type
Malignant BT	Weakly positive	Moderately positive	Strongly positive	Negative	Wild type
HGSC	Negative	Negative	Negative	Positive	Mutant-type

Abbreviation: IHC: Immunohistochemistry, BT: Brenner tumor, HGSC: High-grade serous carcinoma.

**Table 4:** Immunohistochemical characteristics of mucinous ovarian neoplasms.

Line of Differentiation	Immunohistochemical Profile	Percentage
Intestinal	CLDN18+/CDX2±/ER−	71%
Mullerian	CLDN18−/CDX2−/ER+	6%
Indeterminate	CLDN18+/CDX2±/ER+	22%

## Data Availability

Not applicable.

## Supplementary Materials

The following supporting information can be downloaded at: https://www.mdpi.com/article/10.3390/medsci11010018/s1, Supplementary Table S1. Clinicopathological characteristics of patients.
