# Supplementary material for: Brenner Tumor of the Ovary: A 10-Year Single Institution Experience and Comprehensive Review of the Literature
Source: Med Sci (Basel). 2023 Feb 7;11(1):18. doi: 10.3390/medsci11010018 (PMC9944520; doi:10.3390/medsci11010018)
Supplement: Supplementary file 1 [file medsci-11-00018-s001.zip › medsci-2024416-supplementary.pdf]

Table S1. Clinicopathological characteristics of patients.

Clinicopathological variables	Number of patients N
Mode of presentation (n=9)	
Non-incidental	2
Incidental	7
Presenting symptoms (n=9)	
Asymptomatic	1
Postmenopausal bleeding	1
Abdominal distension	1
Abdominal pain	2
Urinary incontinence	1
Abnormal uterine bleeding	1
Recurrent urinary tract infections	1
Abdominal pain and distension	1
Indication of surgery (n=9)	
Bilateral ovarian cysts on imaging	1
Prophylactic surgery (BRCA 1 carrier)	1
Adnexal mass	5
Cervical prolapse	1
Endometrial hyperplasia and fibroids	1
Type of surgery (n=9)	
BSO + Hysteroscopic polypectomy	1
BSO	1
TAH + BSO	3
Exploratory laparotomy + TAH + BSO	1
TVH + BSO	1
Robotic right oophorectomy	1
TAH + BSO and appendectomy	1
Laterality (n=9)	
Right	6
Left	2
Right and left	1
Histologic type (n=9)	
Benign	9
Borderline	0

Malignant	0
Other findings (n=9)	
Mucinous cystadenoma	2
Leiomyomata uteri	3
Corpus luteum cyst	1
Endometrial polyp	2
Walthard rests (n=9)	
Present	6
Absent	3
Transitional metaplasia (n=9)	
Present	4
Absent	5

Abbreviations: BSO: Bilateral salpingo-oophorectomy; TAH: Total abdominal hysterectomy; TVH: Total vaginal hysterectomy
